# Multiple Myeloma Outpatient Transplant Program in the Era of Novel Agents: State-of-the-Art

**DOI:** 10.3389/fonc.2020.592487

**Published:** 2020-11-11

**Authors:** Massimo Martino, Annalisa Paviglianiti, Mara Memoli, Giovanni Martinelli, Claudio Cerchione

**Affiliations:** ^1^ Stem Cell Transplant Program, Clinical Section, Department of Hemato-Oncology and Radiotherapy, Grande Ospedale Metropolitano “Bianchi-Melacrino-Morelli”, Reggio Calabria, Italy; ^2^ Department of Hematology and Cellular Therapy, Saint Antoine Hospital, AP-HP, Paris, France; ^3^ Department of Medicine and Surgery, Hematology and Hematopoietic Stem Cell Transplant Center, University of Naples Federico II, Naples, Italy; ^4^ Hematology Unit, Istituto Scientifico Romagnolo per lo Studio e la Cura dei Tumori (IRST) IRCCS, Meldola, Italy

**Keywords:** multiple myeloma, autologous stem cell transplantation, outpatient, inpatient, novel agents, cost-effectiveness

## Abstract

Multiple myeloma (MM) is the most common indication for autologous stem cell transplantation (ASCT), and outpatient models have been widely developed in this setting. Although numerous studies have demonstrated the safety and feasibility of outpatient ASCT, it is not a routine procedure. Stringent guidelines for patient selection and clinical management, including functional status, caregiver support, and psychological aspects, are essential to identify eligible patients. However, there is still no general agreement on these criteria. Quality of life data are limited and contradictory. There is considerable variability in outpatient transplant models, and there are no randomised studies supporting the use of one over the other. Studies evaluating results in terms of long-term survival, transplant toxicity in comparison with a standard approach are lacking. The procedure is cost-effective within the context of a hospital budget, but an in-depth analysis of the real cost of these programmes has yet to be performed.

## Introduction

Multiple myeloma (MM) is an incurable blood cancer. Considered as a chronic condition, it can be treated to slow its spread.

In last decades, the introduction of bortezomib as first-line therapy have provided considerable improvements in treatment and prognosis of patients with MM.

Although novel agents, including monoclonal antibodies, were recently introduced into clinical practise, high-dose chemotherapy followed by autologous stem cell transplantation (ASCT) remains the standard of care for eligible patients (1–4). High-dose melphalan (HDM) (200 mg/m^2^) is the standard conditioning regimen (5) and the procedure is characterised by a very low transplant-related mortality (TRM) (6).

Healthcare systems are always faced with problems due to the counterbalance between demand and supply. Since request arrives randomly, it can generate waiting lists unless treatment capacity exceeds demand levels. In recent years, a significant increase in hospital waiting lists and times in recent years, causing concerns about the appropriate use of healthcare resource was observed (7).

Several randomised trial have demonstrated the feasibility of outpatient ASCT as an optimal approach to managing hospital length of stay (8–21). This procedure is not feasible in several settings, such as low or middle in-come countries.

The ease of administration of HDM, the relatively low extra-haematological toxicity, and the short duration of neutropenia post-chemotherapy make patients with MM a perfect candidate (5).

## ASCT Outpatient Models

Patients scheduled for ASCT are commonly admitted to Transplant Units on an inpatient basis. In this setting, the central venous catheter (CVC) insertion, HDC administration, ASCT, and supportive care during neutropenia performed in positive-pressure reverse isolation rooms with a hospital stay of 3 to 4 weeks. Several trials have investigated different outpatient models to evaluate the safety, efficacy, and potential cost- saving of reducing hospital stay for patients undergoing ASCT (**Tables 1** and **2**).

**Table 1:** Outpatient Autologous Stem Cell Transplantation Models.

Model	Central venous catheter insertion	High-dose chemotherapy administration	Stem cell infusion	Management of aplastic phase	Comments
**Early Discharge**	Inpatient Clinic	Inpatient Clinic	Inpatient Clinic	Outpatient clinic	The most widely used model worldwide
**Delayed Admission**	Outpatient clinic	Outpatient clinic	Outpatient clinic	Inpatient Clinic	The model does not significantly reduce the duration of hospitalisation and its costs when compared to other models.
**Total Outpatient**	Outpatient clinic	Outpatient clinic	Outpatient clinic	Outpatient clinic	This approach is associated with the shorter stay in hospital
**Mixed inpatient-Outpatient**	Outpatient clinic	Outpatient clinic	Admitted to the Inpatient Unit for stem cell infusion on day 0 for 2 days	Outpatient clinic	This programme was primarily designed and used in Italy. Inpatient stem cell infusion is mandatory to obtain the optimal reimbursement according to the Italian diagnosis-related group (DRG) system
**At-Home**	Inpatient Clinic	Inpatient Clinic	Inpatient Clinic	Outpatient Clinic	The most attractive model for the future

**Table 2:** Clinical studies evaluating the management and outcome of Outpatient Autologous Stem Cell Transplantation in Multiple Myeloma.

Author(Year)	Study Design	Specific eligibility criteria	Regimen	Model	No. of Transplants	No. of readmission%	Reasons for hospitalisation	TRM %	Comments
**Ferrara (** 13 **)**	Prospective	Presence of a caregiver, patient adherence, home within a 45-min travelling distance from the hospital	MEL	EDM	48 (PEG) 113 (G- CSF)	(PEG) 88 (G-CSF) 74	FN and severe mucositis. PEG group 12% G-CSF group 26%	PEG= 0 G-CSF=0.8	The administration of single-dose PEG resulted in similar outcome in terms of safety and efficacy with respect to 8 days of G-CSF.
**Faucher (** 8 **)**	Randomized	Available family caregiver 24 h a day, and living within 45 min driving distance to and from the hospital. Patients not meeting these criteria were not discharged	MEL	EDM, IN	66 (EDOM) 65 (IN)	15 (EDOM) 18(IN)	unknown	0	First randomised study comparing EDOM with standard inpatient ASCT. About 40% of patients in the EDOM arm were not discharged for social or psychological reasons
**Martino (** 15 **)**	Retrospective	Caregiver on a 24-h basis; home within easy reach of the transplantation centre; adequate activities of daily living	MEL	EDM	522	18.8	Fever: 14.6% Mucositis: 1.7% Diarrhoea: 1.7% Arrhythmia:0.4 %; TIA: 0.2% Cutaneous haemorrhage: 0.2%	1	No centre effect was observed
**Paul (** 16 **)**	Retrospective	Availability of Caregiver at home; short distance to transplant centre; patient and physician preference; ECOG≦̸1	MEL	EDM	301 (n=82, ≤4days; n=219,≥5 days)	67	Fever: 87% Inability to maintain hydration: 7% Other: 65%	0	Carefully selected patients were managed with a brief initial hospitalisation and outpatient follow-up, with low morbidity and mortality
**Abid (** 18 **)**	Case-Control study	<65 years of age; newly diagnosed, transplant-eligible MM patients	MEL	EDM, IN	10 (EDM), 11 (IN)	Unknown	FN	0	Findings demonstrated that outpatient ASCT can be considered in Asia in carefully selected patients
***DAM***																		
**Anastasia (** 12 **)**	retrospective		MEL	DAM	123	93		0	The model was not associated with a significant reduction in length of hospital stay
***TOM***
**Kassar (** 10 **)**	Retrospective	Patients with a primary care provider	MEL	TOM	90	58	Fever: 33% No Primary Care Provider:13% Mucositis:6% Other: 6%	0	80% of patients remained neutropenic for 5 days or less, and no patient had neutropenia for more than 7 days. The study confirmed the relatively low extra-haematological toxicity and the short period of post-high-dose melphalan neutropenia
**Gerzt (** 11 **)**	Prospective non- randomised study	Availability of a chaperone or Caregiver with them	MEL	TOM	716	39	Declining performance status, mucositis infection with hemodynamic instability	1.1	Younger patients and those with serum creatinine levels less than 1.5 mg/dl were more likely to complete the programme as outpatients
**Holbro (** 14 **)**	Retrospective	Availability of a caregiver and residence close to the hospital	MEL	TOM	91	84	Fever: 85% Mucositis: 6% Other: 9%	0	The cost savings was $19,522 per patient
**Shah (** 19 **)**	Retrospective. Outpatient versus inpatient ASCT	Patients <70 years old with normal organ function, committed Caregiver and residence within 30 min of the cancer centre were considered for outpatient management	MEL or MEL+BU	TOM	Inpatient *n*=669 Outpatient *n*=377	55	The leading cause (42%) of these admissions was neutropenic fever.	Inpatient= 1.5 Outpatient= 0.3	Patients transplanted as outpatients were significantly younger and more likely to have a HCTCI score <2and creatinine <2. The inpatient group experienced significantly more adverse events. Two-year PFS was significantly longer in the out- patient group. Two-year OS was also longer in the outpatient group.
**Kodad (** 20 **)**	Retrospective	Patients with MM, POEMS syndrome, amyloidosis	MEL	TOM	752	245 (32.6%)		0.4	Low transplant-related mortality. Overall resource utilization significantly lower than that of inpatient ASCT. Model requiring a multidisciplinary approach with close follow-up.
**Yip (** 21 **)**			MEL	TOM	54	100	FN and gastrointestinal toxicity	0	The number of post-transplant admissions and the high complications reported are not in line with other studies in this sector
***MIOM***
**Morabito (2002)**	Retrospective	Psychosocial evaluation to establish skills and compliance of patients and caregivers.	MEL	MIOM	60	56.7	FN	0	Patients were admitted for HPC infusion for 2 days for reimbursement purposes.
***Home-Care***
**Martino (** 17 **)**	Three-arm prospective, non- randomised study	Availability of a caregiver who was willing to stay at home and help, and approval of the home by the medical staff of the SCT Unit	MEL	HC/ED OM/IN	HC=15 EDOM=25IN=40	HC=13 EDOM=8	FN	0	Vey Innovative approach

FN, Febrile neutropenia; EDM, early discharge model; TIA, transient ischemic attack; ECOG, Eastern Cooperative Oncology Group; DAM, delayed admission model; MIOM, mixed Inpatient-Outpatient model; TOM, total Outpatient Clinic; HCTCI, Hematopoietic Stem Cell Comorbidity Index; PFS, progression-free survival: OS, overall survival; HPC, hematopoietic progenitor cell; MEL, melphalan, BU, busulfan; SCT, Stem Cell Transplant; HM, home-care, IN, in-patient.

### Early-Discharge Model

In this model, CVC insertion, HDC administration, and ASCT are carried out in positive pressure reverse isolation rooms, whereas supportive care of the aplastic phase is performed on an outpatient basis.

Ferrara et al. reported outcomes of 28 patients with MM who underwent ASCT using an early-discharge model (EDM). There were no cases of early TRM, and the readmission rate was 36% (9). The same authors described a series of 161 MM patients submitted to ASCT on an outpatient basis and managed post-procedure with either post-transplant single-dose PEG-filgrastim (*n* = 48) or conventional daily granulocyte colony-stimulating factors (G-CSF) (*n* = 113) (13). The conditioning regimen was HDM (140 - 200 mg/m2). Overall, a second hospitalisation was required in 32% of cases (36/161 procedures). There was no difference in the rate of readmissions between the PEG-filgrastim and filgrastim groups (12% vs. 26%, respectively, *p* = 0.06), however the low number of patients prevents to draw firm conclusions.

Faucher et al. carried out the first and only randomised trial to date comparing an EDM with standard inpatient ASCT in 131 patients with MM, lymphomas, or solid tumours. In both arms, high-dose chemotherapy (HDC) was administered, and ASCT performed during hospitalisation. Patients in the EDM arm were discharged on day 0, looked after at home by a caregiver, and followed up on an outpatient basis. The study reported a readmission rate of 86%, mainly during the first week (87% of re-admitted patients) and before haematological recovery (93%). Although safe and feasible, the procedure was highly dependent on economic-social factors. Of the 131 patients, 39% with an indication for HDC was not be discharged early for social or psychological reasons such as lack of a caregiver, living far away from the hospital, or patient’s own request. The study demonstrated that the EDM model was highly dependent on caregivers and that only some patients could benefit from it (8).

Martino et al. analysed the outcome of 382 patients with MM who underwent ASCT in EDM in Italy between 1998 and 2012. Overall, TRM was 1%. A second hospital admission during the aplastic phase occurred in 98 (18.8%) patients. Neutropenic fever (NF) was observed in 161 cases (30.8%) and required readmission in 76. The incidence of grade 3-4 mucositis was 9.6%. In multivariate analysis, independent predictor factors of readmission were fever, grade 3–4 mucositis, and delayed transplantation. No centre effect was observed (*p* = 0.36) (16). In 2015, Paul et al. analysed 301 ASCT procedures carried out for MM, including patients with a ≤ 4-day hospitalisation (*n* = 82) and with a ≥ 5-day stay in hospital (*n* = 219) (17). Amongst the shorter stay patients, 67% required readmission before day + 100. They also had a lower cumulative number of days in hospital than the longer stay group (9 vs.18 day, respectively, *p* < 0.0001), a lower infection rate (22% vs. 46%, *p* < 0.001) and fewer admissions to the Intensive Care Units (0% vs. 5.9%, respectively, *p* = 0.02). The 100- day mortality rate was 1.8% (*p* = 0.6) in the longer stay group, whereas no patients died in the short stay group. Subsequently, an Asian study analysed the efficacy, cost-effectiveness, and safety of EDM ASCT (*n* = 10) compared to inpatient ASCT (*n* = 11) in a MM cohort treated in a single centre with relatively good healthcare resources and easy and prompt access to care due to thanks to its small size and excellent emergency medical services. Mortality was 0%. The authors demonstrated that outpatient ASCT could be considered a viable/valid option in Asia in selected patients. The cost of treatment was significantly lower in the outpatient’s arm than in the inpatient’s arm because of higher hospitalisation-related costs for the latter (19).

### Delayed Admission Model

In this model, first proposed in a study by Anastasia et al., HDC and ASCT were performed on an outpatient basis, whereas supportive care for the aplastic phase is provided positive-pressure reverse isolation rooms. The discharge was planned on day one and scheduled re-hospitalisation on day 5. One hundred and forty-four patients with various haematological and non-haematological malignancies entered the programme. The early discharge was feasible in 86% of cases, and only 5% of discharged patients were re-hospitalised before day 5, mainly due to severe mucositis or fever. The delayed admission model (DAM) did not result in a significant reduction and cost of hospitalisation when compared with other models (12).

### Total Outpatient Model

The total outpatient model (TOM) is associated with the shortest duration of hospitalisation. In this case, conditioning chemotherapy and ASCT are performed on an outpatient basis. After ASCT, patients are followed daily in the Outpatient Clinic during the aplastic phase. The feasibility of TOM was first reported by Gerzt et al. (11) in 716 MM patients submitted to ASCT at the Mayo Clinic in Rochester, Minnesota (US). With a median hospitalisation time of 4 days, the study showed that 39% of patients did not require admission. However, the majority of patients who lived too far away from the Centre to commute every day were forced to find accommodation in local hotels to attend the Outpatient Clinic. This approach may substantially increase the out-of-pocket cost burden for patients.

Using a TOM model, Kassar et al. (10) showed the time and duration of neutropenia after HDM (140–200 mg/m^2^). Nearly two-thirds of patients became neutropenic on day 5 and neutropenia lasted 5 days in 80% of the patients, and 7 days in the remaining 20%.

A retrospective analysis of 91 patients with MM who underwent outpatient ASCT showed that TOM ASCT could be performed safely (14). The majority required hospital admission during the first 100 days. Patient age and creatinine > 2 mg/dl were predictive factors for hospitalisation.

Shah et al. compared outcomes of 1,046 MM patients receiving ASCT as an inpatient procedure (*n* = 669) with those treated as outpatients (*n* = 377) (19). Although over half of the outpatients eventually had to be hospitalised (and thus only 20% of patients completed the procedure as outpatients), the overall incidence of adverse events was far lower than that of the inpatient’s arm, with no difference in TRM. Two-year progression-free survival (PFS) and overall survival (OS) were significantly better in the outpatient group (60% vs. 50%, *p* = 0.005 and 83% vs. 77%, respectively, *p* = 0.01). The differences observed were associated with baseline characteristics of patients. Inpatients were older with more comorbidities and more advanced disease.

Outpatient ASCT for MM is the standard of care at the Vancouver General Hospital, given the increasing volume of patients and longer waiting times. Kodad et al. (20) evaluated the number of patients requiring hospital admission, duration of hospitalisation, patient characteristics, TRM, and OS in 724 patients who underwent ASCT. The majority of patients received HDM, the day 100all-cause mortality rate was 0.9%, and the TRM 0.4%. Yip et al. analysed the outpatient programme in 70 patients with MM who underwent HDC and ASCT in London’s hospital (21). The authors concluded that the outpatient transplant programme was a safe procedure for eligible patients. An innovative care pathway has been established in the Mayo Clinic Stem Cell Transplantation Program (22), reducing day 100 mortality (all-cause) to 0.3%. Patients underwent transplantation with a median hospital duration of 0 days and with only 25% of patients requiring hospitalisation ≥ 5 days.

### Mixed Inpatient-Outpatient Model

The mixed inpatient-outpatient model (MIOM) was used in Italy for the first time (9). Ferrari et al. reported that CVC insertion, fluid infusion, HDM, and supportive care during the aplastic phase were carried out in the Outpatient Clinic. Patients with MM were admitted to the hospital for two days during which ASCT was performed. An inpatient setting was mandatory for the procedure in order to obtain the highest reimbursement according to the Italian diagnosis-related group (DRG) system (23). Clinical outcomes were compared with a retrospective cohort of MM patients traditionally transplanted using an inpatient procedure. Patients in the MIOM programme showed a significant reduction in the length of hospitalisation and no increased toxicity. Overall, 6.7% of patients were not discharged after the ASCT, and, amongst those discharged as planned, 43% were re-hospitalised for a median of 9 days, significantly shorter than 20 days observed for patients undergoing conventional inpatient ASCT.

### At-Home Model

The hospital-at-home is it is a model by which healthcare professionals provide the same level of care at the patient’s home as a traditional hospital model. Martino et al. (17) published the preliminary results of a three-arm, prospective, non- randomised study to evaluate the feasibility and safety of a home-ASCT in 80 patients with MM. In the Home-Care arm (n=15), the patients were discharged the day after the transplant and managed daily at home. The mandatory condition for this type of model, in addition to the consent and availability of a caregiver 24/24 h, was the home no more than 20-min drive from the hospital. Patients who did not have a home near the hospital were discharged to a residential facility the day after the transplant and were treated as Outpatients. There were no cases of TRM and no differences in mucositis rates between the three arms of the study. FN incidence was lower in the outpatient (28%) and home- care cohorts (40%) than in inpatients (75%). Re-hospitalisations were necessary for 8% and 15% of outpatients and home-care patients, respectively, all caused by fever

## General Recommendations

The recommend inclusion criteria for MM Outpatient Autologous Stem Cell Transplantation are summarised in **Table 3**. Supportive care (such as management of nausea and vomiting, hydration, analgesic therapy) should not differ from recommended conventional ASCT guidelines (24).

**Table 3:** Suggested Inclusion Criteria for Multiple Myeloma Outpatient Autologous Stem Cell Transplantation.

Age ≤ 65 years
ECOG ≤ 2
Normal cardiac, lung, liver, and renal function
Absence of advanced disease
Absence of gram-negative MDR pathogens colonisation or infection during the 3 months prior to the scheduled transplant
Severe infection not completely resolved
Signed written informed consent
Availability of a caregiver 24 h/24 h
Detailed SOP for the Caregiver and patient
Distance from house to the hospital ≤ 1 h
Outpatient clinic available 24 h/day or bed reserved in the Transplant Unit
A specific phone line 24 h/24 h

ECOG, Eastern Cooperative Oncology Group; MDR, multidrug resistance; SOP, standard operating procedure.

Antimicrobial prophylaxis for outpatient ASCT should not differ from that required for conventional inpatient ASCT (25–27). Levofloxacin prophylaxis is associated with decreased risk of infection and fever (28), and primary antifungal prophylaxis is not recommended (29). Antiviral prophylaxis is recommended at least up to 3 months after transplantation, or until there is a satisfactory immunological recovery (CD4+ lymphocytes 4200/mmc), as well as *Pneumocystis jiroveci* prophylaxis. The first clinical evaluation should be on day +5 after discharge, and then twice weekly until sustained haematological and clinical recovery. Patients, caregivers, and family members should be adequately trained on the careful monitoring of fever and other infectious signs/symptoms.

International guidelines (30, 31) indicate that G-CSFs should be used as primary prophylaxis after chemotherapy when the risk of FN is > 20%, as happens after HDC and ASCT. Short-acting G-CSFs are the standard molecules for enhancing neutrophil recovery after ASCT but the long-acting G-CSF, pegfilgrastim, can also be used (32), Pegfilgrastim is more useful in an outpatient programme (33) as it is given in single doses, thus facilitating the work of staff and caregivers by reducing the total number of drug administrations needed. Recently, a study provided evidence of the superior efficacy of lipegfilgrastim over short-acting G-CSFs for the prevention of severe neutropenia in an MM ASCT setting (34). Lipegfilgrastim is a new, long-acting, once-per-cycle G-CSF for reducing the duration of neutropenia and the incidence of FN in adult cancer patients treated with chemotherapy. It recently received European Union marketing approval.

## Readmission Criteria After Outpatient ASCT

Criteria for a readmission include severe mucositis unresponsive to outpatient management and/or fever > 38.3°C. In case of illness during neutropenia, patients must be evaluated within 1–2 h and blood pressure, O2 saturation, and vital signs carefully monitored. Patients without symptoms can be followed as outpatients after 6 h of clinical monitoring. The guidelines strongly advise the availability of a 24/7 active phone line to the haematologist on call in the Bone Marrow Transplant Unit (24). The clinical examination can be performed either in the Outpatient Clinic by the general practitioner or in Emergency Department in case of clinical worsening. In either case, immediate feedback can be given to the haematologist on call, and, if needed, oral antibiotic treatment can be started. A detailed standard operating procedure (SOP) in the event of FN should be made available to the patient, Caregiver, and general practitioner. Patient should undergo physical exam, blood cultures, and imaging studies when clinically indicated.

The Multinational Association for Supportive Care in Cancer (MASCC) score (35) could be used to evaluate the risk of chemotherapy-associated complications/febrile neutropenia, but this index has yet to be validated in an ASCT setting. The authors concluded that, although a MASCC score of 21 or less (high-risk patients) could be considered a criterion for rapid readmission, a score of 22 or more was not a sufficient criterion *per se* for defining patients at low risk whose readmission could be delayed (35).

Suggested criteria for readmission are reported in **Table 3**: hemodynamic instability (e.g., tachycardia and low blood pressure), impaired respiratory function (increased respiratory frequency and low oximetry on room air), oliguria, altered mental status and other signs of clinical instability**;** Grade > 2 oral mucositis and diarrhoea**;** colonisation by extended-spectrum beta-lactamases producing Enterobacteriaceae (colonisation by other multidrug resistance (MDR) pathogens); fever persisting after two days of broad-spectrum antibacterial therapy; and low patient compliance*”*.

The use of empiric antibacterial therapy must follow internationally accepted guidelines for patients with FN and haematologic malignancies (36–38). Empiric broad-spectrum antibacterial therapy should be initiated within 1 h of the clinical evaluation, and as soon as the fever workup was completed. Outpatient oral antibiotic therapy (i.e., amoxicillin-clavulanate) can be considered (39), although intravenous antibiotics are preferred and should be chosen in the light of clinical and laboratory findings.

In 2018, a meta-analysis of 1940 patients with MM or lymphoma who underwent ASCT compared the risk of FN in outpatients and inpatients (40). The study showed a lower risk of FN, grade 2-3 mucositis, and septicemia in outpatient ASCT. In 2017, a retrospective study evaluating performance status and hematopoietic cell transplantation comorbidity index (HCT-CI) in 448 patients MM patients undergoing outpatient ASCT reported a lower Karnofsky performance status and higher HCT-CI score in inpatient groups than in outpatients (*p* < 0.004) (41).

## Quality of Life

### The Patient’s Side

Transplant is usually associated with physical and psychological sequelae that can contribute to a dramatic decline in patients’ quality of life (QoL) during the 3–4 weeks of isolation after stem cell infusion. Few trials have focused on patient QoL during an outpatient approach, often with conflicting results. Despite de lack of robust clinical data, the impression is that an outpatient transplant approach is correlated with better patients’ QoL.

Summers et al. reported significantly better emotional well-being and QoL in outpatients than in inpatients, supporting outpatient ASCT approach as an ideal form of care for those with appropriate physical and psychological motivation (42). Conversely, overall QoL was not significantly different between outpatients and inpatients in a cohort of MM patients during the first 30 days after ASCT (43). Schulmeister et al. showed that the QoL decreased immediately post-treatment but then increased to above pre-treatment levels by six months (44). An excellent clinical outcome following ASCT was associated with better QoL and greater satisfaction with care. These studies, however, have a significant limitation in that they were observational, non-randomised studies, and patients could choose the type of transplantation procedure (outpatient or inpatient). Thus, subjective QoL outcomes were influenced by the initial choice.

### The Caregiver’s Side

Caregivers include parents, siblings, children, partners, and friends who play a critical role in the recovery from ASCT and are intimately involved in the patient’s care (45). Foster et al. reported that, when a caregiver was involved during the hospitalisation phase of ASCT, the patient outcome in terms of OS was significantly better than in the group without a caregiver (46). Moreover, the cost-cutting and feasibility associated with the outpatient approach appear to be mediated mainly by the efforts of caregivers whose involvement is needed to decrease the need for hospital readmission (47). The majority of centres offering outpatient ASCTs require the availability of a caregiver 24/7 during the post-ASCT period to take on the many responsibilities traditionally shouldered by professionals (24). Such an agreement involves the total dedication of the Caregiver to the patient, which obviously impacts multiple areas of the carer’s life (45).

Whilst several systematic reviews have evaluated the burden of transplant on caregivers (48, 49), only a few have included caregivers of patients receiving stem cell transplantations (SCTs) in the outpatient setting. Overall, the studies have corroborated existing literature on the experience of a signiﬁcant burden amongst SCT caregivers across the SCT trajectory, highlighting the emotional costs of outpatient ASCT on caregivers and the need to identify caregivers at high risk of strain and distress (45, 50). With these premises, it is essential to design and conduct studies that can critically analyse the emotional burden of the caregivers, and the impact it may have on the clinical outcome of the outpatient transplant (51–53).

## Cost Data

Several trials have shown that outpatient ASCT is cost-effective, mainly because of the shorter duration of hospitalisation (8, 54, 55). Holbro et al. reported a cost savings of $19,522 (Canadian dollars) per outpatient ASCT compared to inpatient procedure, with an annual savings of approximately $740,000 (14). Ghatnekaret al. showed that the major contributor to the total cost of MM treatment was the cost of inpatient care (56).

Clemmons et al. reported a reduction of about US $ 2000 per transplant when a mixed inpatient-outpatient ASCT model was applied (57) with a total annual cost saving of US$ 90,000. Shah et al. showed that the average cost of the procedure was $292,572 and$416,154 for the outpatient and the inpatient transplant group, respectively (19).

In the late 1980s, a tariff-calculating method was created using a diagnosis-related group (DRG) system based on the international classification of diseases, patients’ characteristics as gender and age, presence of comorbidities, diagnosis procedures, and discharge status (23). Italian Regions pay the cost of hospitalisation based on the length of hospital stay and the identified DRG, based on a fixed price (58). The impact of new, costly therapies has made the DRG not the best method to evaluate the actual cost of a health service. Activity-based costing (ABC) is a tool developed to improve efficiency and control costs (59, 60). ABC endeavours to assign values to each activity and source, making it easier to understand and administrate total costs.

The use of the ABC system allows scrutiny of the complete map of activities and the relationships that connect them. Its implementation in healthcare centres has been hypothesised since the early 1990s, and now over 20% of hospitals in the U.S. and Canada use this method (61).

Martino et al. calculated the cost of ASCT in MM patients using the ABC method, and showed a charge of €28,615.15 and €16,499.43, in inpatient and outpatient ASCT, respectively. If we considered that in Calabria Region (south of Italy), the DRG reimbursement for a transplant is €60,000, the estimated cost saving per patient is €31,190.85 for the inpatient approach and €43,306.57 for the outpatient model.

Dunavin et al. using a merged dataset of the Center for International Blood and Marrow Transplant Research (CIBMTR) observational database and Centers for Medicare & Medicaid Services Medicare administrative claims data to analyse reimbursement, service utilisation and patient financial responsibility amongst Medicare beneficiaries in 1640 patients with MM who underwent ASCT in inpatient and outpatient settings (62). Total reimbursement and patient responsibility were analysed for patient and disease characteristics. Of the 1640 patients, 1445 (88%) underwent inpatient ASCT and 195 (12%), outpatient ASCT. The adjusted total mean reimbursement was higher for inpatients than outpatients ($82,368 vs. $46,824, respectively; *p* < 0.0001). Adjusted total mean patient responsibility was $4,736 for inpatients and $6,944 for outpatients (*p*< 0.0001). Within 100 days of ASCT, 107 (55%) of the 195 outpatient recipients had required at least one readmission compared with 348 (24%) of the 1445 inpatients. Reimbursement, service utilisation, and financial responsibility varied on the basis of the ASCT setting.

## Discussion

Outpatient ASCT is feasible and safe with a TRM of 1% for MM, making it an appealing alternative to the standard inpatient ASCT. The popularity of outpatient ASCT is limited by concerns that the lack of protective isolation used during inpatient ASCT could predispose outpatients to a higher risk of toxicities, in particular infections. Although several studies have reported a lower incidence of FN in outpatient ASCT, it has yet to be established as a routine procedure, and many haematologists are still reluctant to adopt this approach. The extensive use of outpatient ASCT models in MM could contribute to making ASCT more competitive, especially when compared with the high cost of some new drugs. Opinion leaders should commit to writing specific reference recommendations/guidelines, and rigorous criteria for patient selection, such as stringent selection criteria with emphasis on functional status, caregiving support, and psychosocial aspects.

The main critical points of the outpatient transplant approach are the following: there are no randomised studies that clearly indicate which model is better than another; there are no studies that have analysed survival outcomes after extended follow-up; the real costs of these programmes still need to be calculated. One could speculate that the outpatient procedure is cost effective in terms of hospital budget, but prospective randomised trials are needed to draw firm conclusions.

Some authors report direct savings of between 10% and 50% that are highly influenced by the release of hospital beds and low readmission rates (14, 54, 55). Data available on QoL are limited and contradictory (42, 43). The majority of centres offering outpatient ASCT call for the availability of a caregiver 24/7, at least for the duration of the aplastic phase. In this way, caregivers spend much of their time with patients, which affects multiple areas of their life. Caregivers must prepare their homes or residential facility to avoid potential infectious complications and are responsible for the administration of medications, monitoring of vital signs, and intake and output of fluids, tasks traditionally carried out by professionals. Caregivers of outpatient ASCT patients may also be required to facilitate daily visits to the Outpatient Clinic. Some studies have shown that OS is significantly better in ASCT patients with a caregiver. Therefore, feasibility and safety of an outpatient approach appears to be mediated in large part by the efforts of caregivers. Nevertheless, the lack of a caregiver is the most common reason for a patient’s refusal to take part in an outpatient programme, and this should be considered a bias. The last crucial point is that the majority of trials were not randomised, controlled trials. The characteristics of the patients were different across the groups, and sometimes the decision for an outpatient or inpatient approach was according a subjective physician opinion. This means that the observed difference in the risks of infection may have been a consequence of the different baseline characteristics rather than the effect of the treatment strategy. The eligibility criteria of some trials indicated that patients in the outpatient group were required to have good performance status, no organ failure, and an age ≤65 years. This may have introduced a bias in the form of a selection of only healthier subjects for the outpatient arm. Furthermore, patients could choose the type of transplantation procedure (outpatient or inpatient), and outcomes may thus have been influenced by this choice.

In conclusion, outpatient ASCT for MM is a safe and feasible approach and should be considered by healthcare providers. Given that it is difficult to carry out randomised trials in this setting, rigorous selection criteria are mandatory for the routine use of the outpatient approach. Caregivers play a crucial role in the success of the outpatient procedure. Useful tools to assess the QoL of patients and caregivers are needed to evaluate this aspect of care.

## Data Availability Statement

The datasets generated and/or analysed during the current study are available from the corresponding author on reasonable request.

## Author Contributions

Conceptualization, methodology, design, and writing: MMa, AP, MMe, GM, and CC. Supervision: MMe, CC, and GM. All authors contributed to the article and approved the submitted version.

## Conflict of Interest

The authors declare that the research was conducted in the absence of any commercial or financial relationships that could be construed as a potential conflict of interest.
